# Correlation Between Endocrine and Other Clinical Factors with Peripapillary Retinal Nerve Fiber Layer Thickness After Surgical Treatment of Pediatric Craniopharyngioma

**DOI:** 10.3390/biomedicines14010239

**Published:** 2026-01-21

**Authors:** Agnieszka Bogusz-Wójcik, Klaudia Rakusiewicz-Krasnodębska, Wojciech Hautz, Maciej Jaworski, Paweł Kowalczyk, Elżbieta Moszczyńska

**Affiliations:** 1 Department of Pediatric Endocrinology and Diabetology, Children’s Memorial Health Institute, 04-730 Warsaw, Poland; 2 Department of Pediatric Ophthalmology, Children’s Memorial Health Institute, 04-730 Warsaw, Poland; 3 Department of Clinical Biochemistry, Children’s Memorial Health Institute, 04-730 Warsaw, Poland; 4 Department of Pediatric Neurosurgery, Children’s Memorial Health Institute, 04-730 Warsaw, Poland

**Keywords:** retinal nerve fiber layer, optic chiasma, compressive optic neuropathy, optic coherence tomography, craniopharyngioma

## Abstract

**Background**: Visual dysfunction resulting from damage to the optic nerve and retinal neurons represents a significant concern in the postoperative management of childhood-onset craniopharyngioma (CP) survivors. The study aims to evaluate the influence of clinical parameters assessed in patients before and after neurosurgery of CP on peripapillary retinal nerve fiber layer (RNFL) thickness results, using optical coherence tomography (OCT) as early markers of compressive neuropathy. **Methods**: This study retrospectively examined 73 eyes from 38 individuals diagnosed with CP and 64 eyes from 32 healthy controls matched for age and sex. All patients in the study group underwent a complete endocrine examination before and after surgery. Moreover, all participants in both groups underwent a thorough ophthalmological examination and OCT imaging. The average RNFL thickness was analyzed, along with the RNFL in the superior and inferior sectors and in eight peripapillary sectors around the optic nerve. Clinical variables were analyzed to assess how they relate to alterations in RNFL thickness within specific sectors. **Results**: After surgery, the peripapillary RNFL thickness was much lower in the CP group than in the healthy control group. Preoperative factors significantly affecting RNFL reduction are as follows: age below 5 years at the time of diagnosis, birth in the country, optic disc oedema, delayed puberty, arginine vasopressin deficiency (AVD), growth hormone deficiency (GHD), hyperprolactinemia, and the degree of preoperative hypothalamic involvement. Moreover, syndrome of inappropriate secretion of antidiuretic hormone (SIADH), as well as the end of AVD, memory disorder and hyperfagia after surgery, correlated with damage to RNFL. **Conclusions**: CP causes significant thinning of the RNFL, which demonstrates the tumor’s impact on the visual pathway. Monitoring optic nerve damage and assessing outcomes after surgery can be performed effectively using OCT. Additionally, the relationship between RNFL thickness in specific areas and clinical indicators can provide vital information for diagnosing and monitoring. This highlights their usefulness in forecasting visual results. As a result, ongoing RNFL assessments should be part of the long-term management of CP patients to improve visual outlook and identify ongoing or remaining damage.

## 1. Introduction

Childhood-onset craniopharyngioma (CP) is a rare and slow-growing epithelial brain tumour (World Health Organisation grade I) [1]. It is thought to arise from embryonic remnants of Rathke’s pouch, located along the craniopharyngeal duct. CP is commonly located in the sellar and/or suprasellar region of the brain. The incidence of CP is 0.5–2 cases per million people per year. Due to the benign histology of the tumour, a 10-year survival rate of 90% has been reported in children [2].

CP is one of the most challenging types of childhood central nervous system tumours due to the long-term sequelae caused by the proximity of CP to the optic nerves, optic chiasm and hypothalamic-pituitary axes [2,3,4,5,6,7].

Due to variability in tumour location, growth pattern, size, and suprasellar extension, CP patients present with diverse symptoms at the time of CP diagnosis. Visual impairment is one of the most frequent presenting manifestation, affecting 62–84% of patients at the time of CP diagnosis [3,8], alongside increased intracranial pressure and hormonal deficiencies.

Patients with CP may experience reduced visual acuity, visual field disturbances like bitemporal hemianopia, or optic disc swelling as a result of the tumor itself or interventions used to treat it [9]. Such visual challenges can greatly affect a person’s ability to perform everyday tasks and diminish quality of life following CP therapy [8]. Importantly, the condition of vision at the time of diagnosis plays a crucial role in predicting long-term visual results during follow-up after tumor therapy [10]. In pediatric CP patients, persistent visual disturbances after surgery occur in 48% to 75% of cases [11]. Visual impairment most commonly results from the expansion of a suprasellar tumor that physically compresses the optic chiasm and nerves. Persistent pressure can cause permanent injury to optic nerve cells, such as retinal ganglion cells and their fibers, resulting in a thinner retinal nerve fiber layer (RNFL). As a result, affected individuals may experience a range of vision problems, including decreased visual acuity, loss of visual fields, and swelling or atrophy of the optic disc [4,12,13,14,15,16,17,18,19]. Timely detection and ongoing observation of these visual issues are essential, since the extent of optic nerve injury frequently aligns with both the seriousness of vision loss and the likelihood of recovery after surgery [17,20,21,22,23].

Structural damage may be detectable before vision loss is diagnosed by standard automated perimetry, a test that measures the visual field. Perimetry in children is technically challenging, often yielding nondiagnostic results and making accurate interpretation difficult. Optical coherence tomography (OCT), an imaging technique that provides objective and quantitative measurements of the optic nerve and retinal layers, is more accurate, easier to perform, and less affected by variability in patient cooperation, thereby improving diagnostic accuracy and reproducibility [21]. As a result, simpler, faster diagnostic tests are being sought that can objectively and indirectly detect changes in the optic nerve in this patient group [24]. Consequently, OCT serves as a practical and reliable diagnostic technique for children. For patients with CP, OCT has proven to be an essential approach for evaluating optic nerve damage prior to and following surgery, as well as for ongoing monitoring and follow-up care [18,19,25,26]. Yet, the link between optic nerve compression and RNFL thinning in pediatric CP cases remains unclear. Various factors—such as the size and form of the tumor, degree of chiasmal compression, and the specifics of surgical intervention—may influence how severely the optic nerve is affected [27]. Furthermore, hormonal and hypothalamic influences may play a role in postoperative results [12,13,28,29]. For CP patients treated with radiation therapy, vision problems necessitated close medical monitoring, since radiation exposure can harm the visual pathways and result in complications like optic neuropathy caused by radiation, retinopathy, necrosis of the visual pathways, and ocular toxicity [30,31].

This study investigated how clinical factors, both before and after surgery related to optic chiasm compression, affect RNFL thickness in pediatric CP patients. For the first time, the impact of tumour clinical factors on RNFL damage was analysed in a large group of these patients. This research clarifies the key mechanisms of optic nerve damage and the relationships between clinical parameters, informing treatment optimisation, monitoring, and ultimately supporting a better quality of life for affected children.

## 2. Materials and Methods

This single-centre, observational, retrospective, cross-sectional study involved patients with early-onset CP who received treatment at the Children’s Memorial Health Institute in Warsaw, Poland, between June 2021 and September 2024. Participants qualified for the study if they were younger than 18 years and had a sellar tumor detected by pituitary MRI, regardless of chiasmal compression, that was surgically treated and histopathologically confirmed as adamantinomatous CP. The Institutional Bioethics Committee of the CMHI in Warsaw (21/KBE/2024; 19 June 2024) approved the study, which was conducted in accordance with the Declaration of Helsinki. All participants aged 13 or above, as well as the legal guardians of those younger than 13, gave written informed consent after receiving comprehensive information about the study’s protocol, objectives, and potential risks.

A total of 73 eyes from 38 pediatric patients (22 males and 16 females) who received neurosurgical treatment for craniopharyngioma (CP) were enrolled in the study. The mean age at OCT assessment was 10.3 ± 4.2 years, with a range of 4 to 17 years.

These patients received care in the Endocrinology Department, and their eye examinations were performed by the Ophthalmology Department at the Children’s Memorial Health Institute. The study group was limited to patients who had adamantinomatous CP confirmed by postoperative histopathology. The control group comprised 64 eyes from 32 healthy children (12 males and 20 females) with no history of CP or other systemic diseases, matched for both age and sex. Their mean age at OCT testing was 10.5 ± 3.1 years (range 4–17).

Participants were not eligible for inclusion if they had any ocular disease, prior eye surgery, or coexisting conditions such as glaucoma, hereditary retinal dystrophies, optic nerve disorders, retinal disorders, significant corneal or lens opacities, macular disease, or a previous glaucoma diagnosis. Those with refractive errors exceeding 3 diopters (D) were also excluded. Both groups were further screened for systemic illnesses, including previously diagnosed and managed conditions like diabetes mellitus, isolated hypertension, kidney disease, neurological disorders, prematurity, or any other disorder known to impact the RNFL. All subjects in both the study and control groups underwent comprehensive ophthalmic assessment, which included best-corrected visual acuity (BCVA), slit-lamp examination of the anterior segment, and fundoscopy after dilating the pupil with 1% Tropicamide.

**Figure 1:**
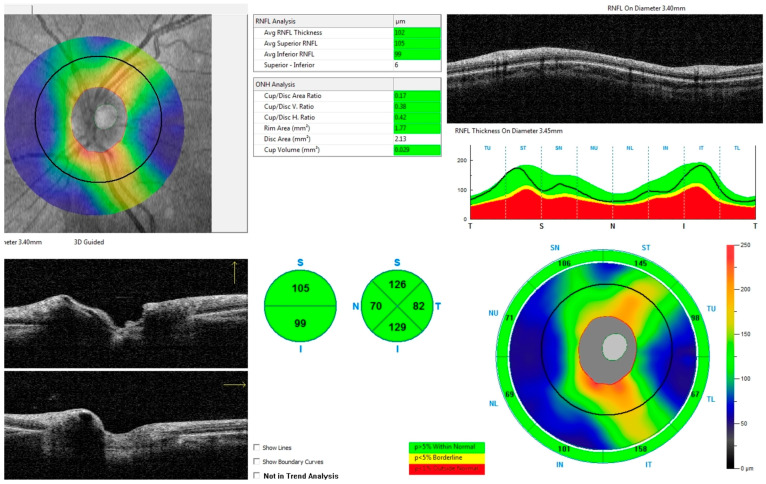
Peripapillary RNFL thickness analysis using spectral-domain OCT. The RNFL thickness map (top left), circular B-scan (top right), and RNFL deviation map (bottom right) show sectoral RNFL measurements with color-coded normative comparison (green: within normal limits, yellow: borderline, red: outside normal limits). Two horizontal OCT B-scans acquired through the peripapillary region and the optic nerve head, illustrating the RNFL profile along the scan path (bottom left).

**Figure 2:**
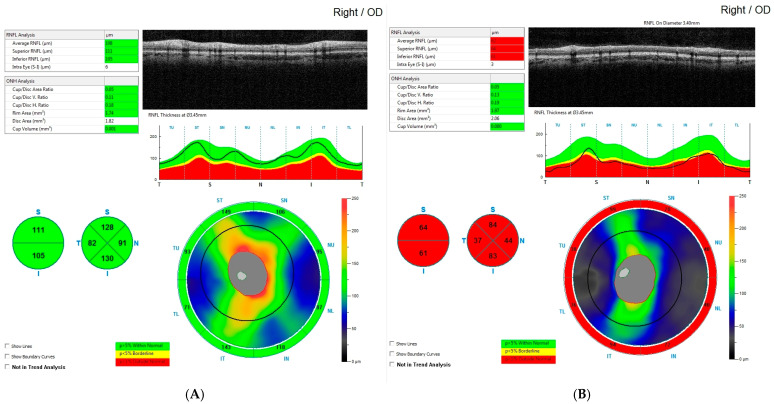
Figure showing retinal nerve fiber layer in the CP group: (**A**) a patient from the control group with normal results, and a patient from the study group with RNFL thinning following neurosurgery for craniopharyngioma (**B**).

All individuals in both groups underwent optical coherence tomography (OCT) using the RTVue XR Avanti with AngioVue (Optovue, Fremont, CA, USA). The scans included in this study were performed 1.5 to 5 years after surgery—an interval during which RNFL thickness had stabilized and stopped declining. The mean duration between surgery and OCT was approximately 3 years and 8 months. Each scan followed the ONH (optic nerve head) protocol. Measurements were based on the protocol’s automated segmentation, which evaluates peripapillary thickness within a 4.0 mm circle. The ONH protocol also provides average peripapillary retinal nerve fiber layer (avgRNFL) thickness, as well as separate measurements for the superior (supRNFL) and inferior (infRNFL) segments (see Figure 1). Additionally, RNFL thickness was measured in eight peripapillary sectors: in the superior hemiretina—Temporal Upper (TU), Superior Temporal (ST), Superior Nasal (SN), Nasal Upper (NU); and in the inferior hemiretina—Nasal Lower (NL), Inferior Nasal (IN), Inferior Temporal (IT), and Temporal Lower (TL) (see Figure 2).

All study group participants underwent detailed assessments before and after undergoing surgery. The pre-surgical assessments covered these parameters: gender, place of birth, age at CP diagnosis, the reason for initiating the diagnostic procedure, and presenting symptoms at diagnosis, such as headache, vomiting, impaired visual acuity, visual field restriction, optic nerve disc oedema, optic nerve disc atrophy, strabismus, double vision, unilateral blindness, drowsiness or altered consciousness, apathy, epileptic seizures, loss of consciousness, memory disturbances, and other neurological symptoms. Endocrinological parameters analyzed at the time of diagnosis included body mass index standard deviation score (BMI SDS), growth retardation, delayed puberty, growth hormone deficiency (GHD), hypothyroidism, adrenal insufficiency, arginine vasopressin deficiency (AVD), and hyperprolactinemia. Tumor characteristics were evaluated, encompassing volume, maximum diameter, solid or cystic morphology, location (intrasellar and suprasellar or suprasellar or intrasellar), calcifications, invasion of the third ventricle, ventriculoperitoneal shunt, preoperative hypothalamic involvement, cavernous sinus infiltration, hydrocephalus, and the presence of a ventriculoperitoneal shunt.

Postoperative evaluation considered factors such as the type of surgical approach (bifrontal craniotomy, transcortical-transforaminal craniotomy), extent of tumor removal (gross total or subtotal resection), histopathology results, detection of Rosenthal fibers, the need for reoperation due to recurrence or progression, the reason for reoperation, and the use of radiotherapy. Rosenthal fibres are detected in microscopic examination of the tumour; this is the glial tissue’s response to the presence of the tumour. Recurrence referred to the return of a tumor after it had been entirely removed, as verified by postoperative MRI or CT scans. Progression described the enlargement of any remaining tumor tissue after a partial removal, regardless of the presence of clinical symptoms, and necessitating additional treatment. After surgery, all patients developed hypopituitarism, and their need for hormone replacement therapy was evaluated. During follow up we analysed the end of AVD, memory disorder and hyperfagia. Comprehensive information about the study group can be found in Table 1 and Table 2.

## 3. Statistical Analysis

Statistical analysis was conducted using Statistica version 10 (StatSoft Inc., Tulsa, OK, USA). To check whether the analyzed variables followed a normal distribution, the Shapiro–Wilk test was employed. Categorical variables are reported as numbers and percentages, while continuous variables are expressed as medians with interquartile ranges (IQRs) and overall ranges (minimum to maximum). The Mann–Whitney test was used for comparisons between two groups, and non-parametric ANOVA with post-test when comparing more than two groups. Correlations were analyzed using Spearman’s R-value. Statistical significance was set at *p* < 0.05.

## 4. Results

The analysis included 73 eyes from 38 children diagnosed with CP (average age 10.3 ± 4.2 years, ranging from 4 to 17; 22 boys and 16 girls) and 64 eyes from 32 healthy controls of similar age and gender (average age 10.5 ± 3.1 years, range 4–17; 12 boys and 20 girls). The average age at the time of CP diagnosis was 8.5 ± 3.9 years (range 1.9–16). Compared with healthy controls, CP patients exhibited significantly thinner peripapillary RNFL in all measurements. Specifically, avgRNFL (79 μm, range 52–117 vs. 106 μm, range 93–137, *p* < 0.001), supRNFL (81 μm, range 50–120 vs. 107 μm, range 93–158, *p* < 0.001), and infRNFL (77 μm, range 49–120 vs. 102 μm, range 90–128, *p* < 0.001) were markedly reduced in the CP group after neurosurgical treatment. Significant thinning was also observed across nearly all individual RNFL quadrants: ST (117 μm vs. 141 μm, *p* < 0.001), TU (60 μm vs. 89 μm, *p* < 0.001), TL (51 μm vs. 89 μm, *p* < 0.001), IT (113 μm vs. 147 μm, *p* < 0.001), IN (87 μm vs. 114 μm, *p* < 0.001), NL (56 μm vs. 77 μm, *p* < 0.001), NU (61 μm vs. 88 μm, *p* < 0.001), and SN (85 μm vs. 113 μm, *p* < 0.001). These results indicate widespread RNFL loss in CP patients compared with healthy peers.

The most frequent reasons for requesting diagnostics were headache (17 patients, 45%), visual impairment (10 patients, 26%), and short stature (4 patients, 11%). The most common presenting symptoms at diagnosis were headache (26 patients, 68%), followed by visual impairment (22 patients, 58%), and growth retardation (16 patients, 42%). In 10 patients (19 eyes, 26%) in whom visual impairment was the cause of initiating diagnostics, the history of symptoms lasted a median of 13 weeks (range: 2 weeks–5.5 months) before the CP diagnosis. On the ophthalmologic examination at the CP diagnosis, impaired visual acuity was found in 41 eyes of 22 patients (58%). The field of vision was checked in 23 patients before CP diagnosis, and restriction was identified in 14 eyes of 7 patients (30%). Optic nerve discs oedema was present at CP diagnosis in 22 eyes (30%), atrophy of the optic nerve disc was found in 7 eyes (10%), strabismus in 17 eyes (23%), double vision in 4 eyes (5%), and blindness of one eye in 2 patients (5%). The median BMI SDS at the time of CP diagnosis was 0.79 SDS (range: −1.33 to +5.24). In 24 patients (46 eyes, 62%), the tumor was located in both intrasellar and suprasellar regions, and in 14 patients (27 eyes, 37%). It was confined to the suprasellar region. The median initial tumor volume was 35.2 cm^3^ (range: 1.8–213.7 cm^3^). The median maximum diameter was 44.2 mm (range: 19–98 mm). Four patients had solid and 34 patients cystic CP (89%). At CP diagnosis, 28 CP patients (53 eyes, 88%) presented with preoperative hypothalamic involvement [32], which a neuroradiologist confirmed.

After surgery, syndrome of inappropriate secretion of antidiuretic hormone (SIADH) occurs in 20 patients (53%). CP progression was found in 5 patients (13%) and recurrence in 4 patients (11%). Seven of them were reoperated on. Nineteen eyes of 10 CP patients (24%) underwent postoperative irradiation. The end of AVD was confirmed in 4 patients (11%). During patient follow-up, memory disorders were found in 14 patients (37%), while hyperphagia was found in 19 patients (50%). Comprehensive information about the study group can be found in Table 1 and Table 2.

**Figure 3:**
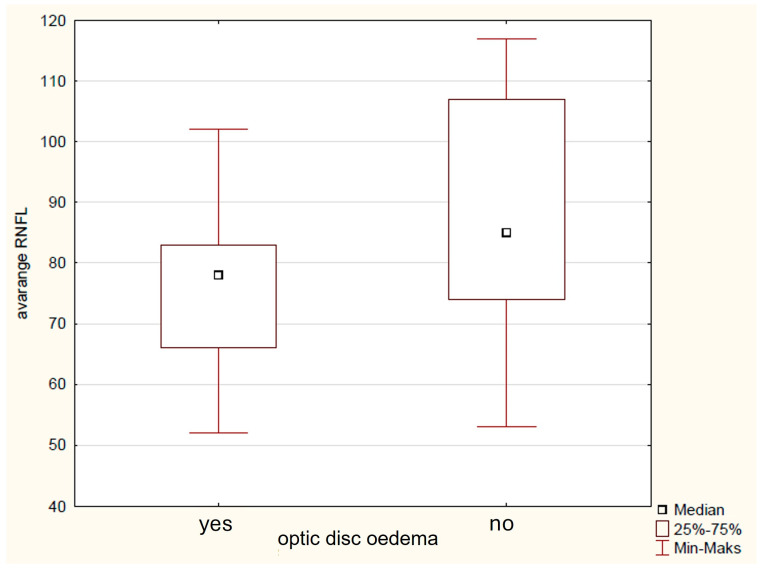
Graph of the influence of the presence of optic disc oedema on the average RNFL thickness.

**Figure 4:**
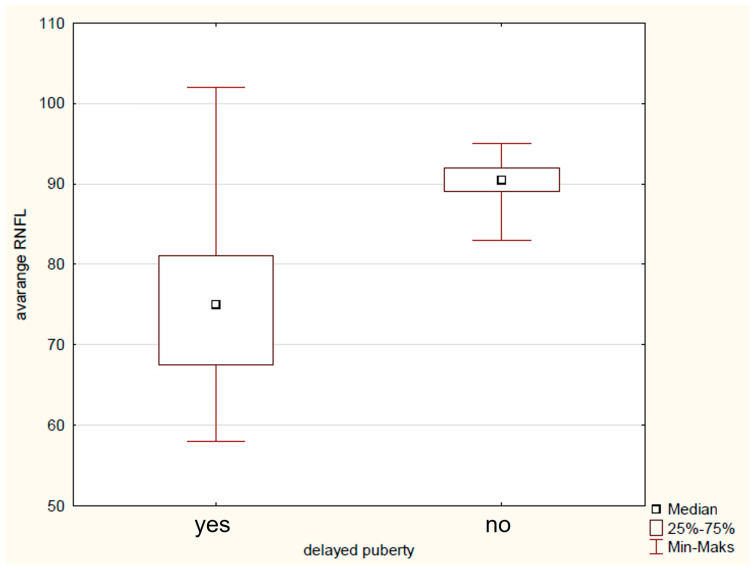
Graph of the influence of delayed puberty on the average RNFL thickness.

**Figure 5:**
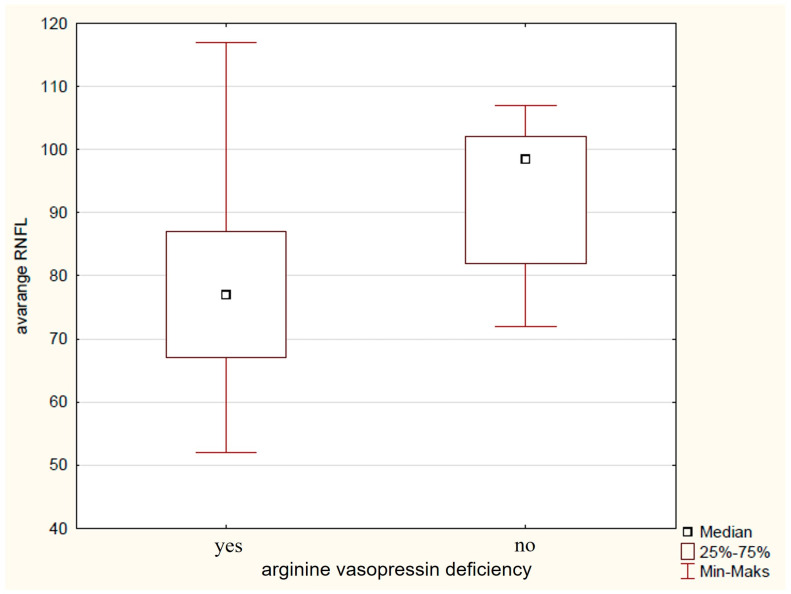
Graph of the influence of arginine vasopressin deficiency on the average RNFL thickness.

Preoperative clinical factors, such as the age below 5 years at diagnosis, were found to significantly influence RNFL damage in the IN (79 μm vs. 121 μm, *p* = 0.02) and NU (56 μm vs. 96 μm, *p* = 0.02) sectors, with more pronounced damage seen in children diagnosed before age 5. Children born domestically exhibited thinner RNFL and showed greater reductions, especially in the infRNFL (74 μm vs. 85 μm, *p* = 0.03), IT (112 μm vs. 130 μmp = 0.03), IN (78 μm vs. 130 μmp = 0.04), NU (29 μm vs. 37 μmp = 0.04), and SN (79 μm vs. 90 μm, *p* = 0.004) regions. Additionally, the presence of optic disc oedema before surgery was significantly linked to avgRNFL (75 μm vs. 85 μm, *p* = 0.02) (Figure 3), supRNFL (78 μm vs. 87 μm, *p* = 0.03) infRNFL (74 μm vs. 83 μm, *p* = 0.03), IN (91 μm vs. 112 μm, *p* = 0.04), and NL (51 μm vs. 62 μm *p* = 0.04) thinning, indicating that optic disc oedema corresponded to more severe RNFL fiber loss in these areas. Delayed puberty before diagnosis was linked to increased RNFL thinning in several areas, including avgRNFL (75 μm vs. 91 μm, *p* = 0.005) (Figure 4), supRNFL (77 μm vs. 92 μm, *p* = 0.007), infRNFL (74 μm vs. 90 μm, *p* = 0.007), ST (109 μm vs. 129 μm, *p* = 0.03), TU (62 μm vs. 78 μm, *p* = 0.007), IT (101 μm vs. 135 μm, *p* = 0.007), NL (52 μm vs. 67 μm, *p* = 0.02), and SN (81 μm vs. 91 μm, *p* = 0.02). Growth hormone deficiency (GHD) detected before surgery also showed a significant association with thinner RNFL measurements in avgRNFL (73 μm vs. 96 μm, *p* = 0.03), infRNFL (66 μm vs. 91 μm, *p* = 0.03), IN (63 μm vs. 122 μm, *p* = 0.03), NL (49 μm vs. 72 μm, *p* = 0.03), NU (58 μm vs. 83 μm, *p* = 0.03), and SN (83 μm vs. 127 μm, *p* = 0.03), with GHD presence indicating more severe RNFL loss in these sectors. AVD diagnosed before surgery significantly influenced the thickening of avgRNFL (77 μm vs. 98 μm, *p* = 0.02) (Figure 5), supRNFL (78 μm vs. 87 μm, *p* = 0.04), infRNFL (76 μm vs. 95 μm, *p* = 0.006), IN (78 μm vs. 119 μm, *p* = 0.04), NU (57 μm vs. 78 μm, *p* = 0.04), and SN (81 μm vs. 123 μm, *p* = 0.008). Hyperprolactinemia before surgery was associated with more pronounced RNFL thinning in the infRNFL (66 μm vs. 78 μm, *p* = 0.02), IN (64 μm vs. 80 μm, *p* = 0.03), and NL (43 μm vs. 53 μm, *p* = 0.003). Moreover TL (34 μm vs. 50 μm, *p* = 0.001) and NL (42 μm vs. 56 μm, *p* = 0.04) were thinner when there was no hypothalamic involvement [32].

Clinical factors after surgery, such as SIADH, had a significant impact on the greater damage in NL sector (49 μm vs. 61 μm, *p* = 0.01). Additionally, the end of AVD in follow-up had a substantial effect on the IT sector. (98 μm vs. 115 μm, *p* = 0.04). A thinner RNFL was observed when AVD was continuous after surgery. Postoperative clinical symptoms such as memory impairment had a significant impact on the RNFL reduction in the TL sector (55 μm vs. 48 μm, *p* = 0.03) as well as hyperphagia in the IN sector (73 μm vs. 90 μm, *p* = 0.002). The detailed impact of clinical symptoms before and after surgery on RNFL damage is presented in Table 3. Clinical parameters before surgery, such as gender, headache, vomiting, impaired visual acuity, visual field restriction, optic nerve disc atrophy, strabismus, double vision, unilateral blindness, drowsiness, altered consciousness, apathy, epileptic seizures, loss of consciousness, abdominal pain, and memory disturbances, did not affect RNFL parameters. Endocrinological parameters like BMI SD, growth retardation, hypothyroidism and adrenal insufficiency had no significant impact on RNFL parameters in individual sectors.

## 5. Discussion

Our study demonstrates that CP is associated with widespread thinning of the peripapillary RNFL across all quadrants, highlighting the substantial impact of the tumor and its treatment on the optic nerve. Compared with healthy, age- and sex-matched controls, CP patients exhibited significant reductions in avgRNFL, supRNFL, infRNFL, and all individual RNFL sectors, consistent with previous reports indicating that compressive optic neuropathy is a significant cause of visual morbidity in this population [12,14,16,23].

The high prevalence of visual disturbances at diagnosis impaired visual acuity in 58% of eyes, visual field defects in 30%, and optic disc oedema or atrophy in a notable proportion of patients, underscoring the vulnerability of the optic pathways to tumour-related compression [31]. The fact that visual symptoms preceded diagnosis by several weeks in some patients highlights the importance of early detection and the potential role of routine ophthalmologic screening in children presenting with headache, growth retardation, or other subtle neurological signs [12]. We identified significant risk factors for RNFL damage in children with CP, both before and after surgery.

This study is the first to evaluate various clinical factors and their influence on RNFL in children after neurosurgery for CP. In CP patients, optic nerve damage may result from direct compression, persistent optic disc swelling, surgical intervention, or radiation therapy [24]. Compression of the optic chiasm is regarded as the primary factor responsible for neuronal cell injury and thinning of the RNFL in sellar tumors. Retrograde degeneration was recognized as the mechanism leading to retinal layer thinning seen in cases of chiasmal compression and other optic nerve disorders [33]. Our findings also identify clinical parameters that influence RNFL thinning, a discovery not previously reported.

Thinner RNFL measurements were associated with younger age at diagnosis, suggesting that the developing visual system in younger patients may be more vulnerable to tumour- and surgical-intervention-related damage. Additionally, children under five years of age often struggle to communicate subtle clinical symptoms. Research in healthy populations consistently demonstrates an inverse relationship between RNFL thickness and age: younger individuals generally have thicker RNFL layers, whereas RNFL thickness naturally thins with ageing [34]. The pronounced RNFL thinning seen in younger patients highlights the critical need for early intervention to protect optic nerve cells in cases of CP. This observation supports the hypothesis that the immature visual system in children is especially susceptible to damage from optic pathway compression and subsequent surgical procedures [35,36].

In our study, we observed a correlation between the presence of optic disc oedema and the severity of RNFL damage, as patients with papilloedema demonstrated a statistically significant reduction in RNFL thickness. Optic disc oedema induces secondary changes in the retinal nerve fibre layer, primarily through mechanical compression of axons and disruption of axoplasmic transport [37]. In the early stages, OCT often shows marked RNFL thickening, reflecting axonal oedema and fluid accumulation [38,39,40]. However, with chronic oedema, progressive axonal loss develops, resulting in RNFL thinning and irreversible retinal ganglion cell damage [41,42]. The extent and progression of these changes depend on both the underlying aetiology and the duration of oedema, with conditions such as persistent intracranial hypertension or chronic inflammation frequently leading to permanent structural damage. In our study group, RNFL measurements were not performed during the acute phase but after neurosurgery, when the swelling had resolved and optic nerve atrophy was already present. The results of our study are consistent with these assumptions and align with findings from previous research [43,44,45].

This study found that children with delayed puberty, hyperplactinemia, GHD, AVD before diagnosis had worse outcomes in RNFL thickness. The major RNFL damage observed in these cases can be attributed primarily to the greater volume and closer proximity to the optic pathway, as presented in our previous study [27]. Larger tumour volume and diameter correlated with greater damage to the RNFL [27]. Ogmen et al. [46] studied the thickness of chorioretinal layers in patients with prolactinoma. The mean RNFL thickness was thinner in patients with prolactinoma than in the control group (*p* < 0.05). None of the patients had a visual field defect. The thickness of retinal layers was similar in patients with and without a complete biochemical response to treatment (*p* > 0.05). Danesh-Meyer et al. reported that the RNFL was thinner in 15% of 40 patients before surgical resection of parachiasmal tumours, although there were no visual field defects [18]. It is explained that compressive damage to the anterior visual pathway may have occurred before the onset of an apparent visual field defect. In all these studies, thinning of the RNLF was attributed to chiasmal compression. Nalcacioglu-Yuksekkaya et al. demonstrated that the mean RNFL thickness in children with congenital isolated GHD was statistically significantly thinner than in healthy subjects (*p* < 0.05). This indicates that GH plays an essential role in the development of the neural retina [47]. What is more, Baudet et al.l suggest that GH acts as an autocrine or paracrine signaling molecule to promote axon growth in a developing nervous tissue, the neural retina of chick embryos [48]. The impact of AVD and gonadotropin deficiency on RNFL thinning has not been thoroughly investigated and described in detail in the literature. It seems likely that the pressure exerted by the tumour on the pituitary gland and hypothalamus is associated with the destruction of these structures and the failure to secrete pituitary hormones. The size and location of the tumour are important markers of the chronicity of optic pathway compression [27]. It is also worth noting that the surgical procedure itself can lead to damage to nerve fibres, which is why a multifactorial assessment of the impact of clinical factors on the RNFL before and after surgery is essential. This study is currently being conducted at the CMHI in Warsaw, Poland. Identifying clinical factors for RNFL damage has practical implications in everyday clinical practice with children.

Precise assessment of visual function and early identification of visual issues contribute to better patient outcomes and enhanced quality of life for children with CP. Additionally, handheld OCT devices make it possible to carry out optical coherence tomography effectively even in young children who may not cooperate fully.

In contrast, visual acuity and visual field testing are less reliable because they require the patient’s cooperation. Table 4 presents the proposed standard of ophthalmological care for patients with craniopharyngioma used at Children’s Memorial Health Institute in Warsaw, Poland. Consequently, early detection of potential ocular damage will inform treatment decisions and facilitate timely referral to visual rehabilitation centres.

It is important to recognise several limitations of this study. Firstly, the retrospective design may have led to selection bias. Secondly, although the sample size was sufficient for initial analysis, it may restrict the broader applicability of the results. The interval between surgery and examination was relatively long; therefore, it cannot be excluded that additional factors may have influenced the RNFL parameters during this period, as well as the direction and magnitude of these effects. In addition, uncooperative patients—primarily the youngest children—or those who were unable to maintain stable fixation due to severely reduced vision were not included. Consequently, OCT assessments were not possible in the most severely affected cases, potentially introducing selection bias by omitting eyes with the most significant structural damage.

There is a lack of detailed studies in the literature on the effect of endocrine abnormalities, such as AVD and gonadotropin deficiency, on RNFL; further studies are needed to assess this relationship.

This study highlights OCT’s importance as a non-invasive and objective method for evaluating optic nerve in children with CP. Measuring RNFL thickness helps clinicians identify subtle lesions at an early stage, making it especially useful for pediatric patients. OCT can also support ongoing monitoring to detect those at risk of vision loss and inform targeted treatment strategies. Retinal nerve fiber evaluation would be a helpful tool both preoperatively and immediately postoperatively in prognosticating long-term visual function.

## 6. Conclusions

In conclusion, CPs are the most common suprasellar region tumours in children. They can cause systemic complications and ocular consequences. Long-term follow-up of a patient’s vision is necessary, even when the patient is being treated and has no visual field defect, due to potential ocular complications arising from the treatment. Our study presents new data that cannot be easily explained by a single factor due to its multifactorial nature.

Overall, our findings reinforce the importance of early diagnosis, careful pre- and postoperative ophthalmological assessment, and consideration of endocrine and neurological factors in the management of pediatric CP. Integrating regular RNFL evaluation using OCT into long-term follow-up protocols may optimise visual prognosis and provide an objective tool to monitor disease progression or recovery after treatment. Patients after CP treatment require multidisciplinary specialist care, including ophthalmological care.

## Figures and Tables

**Table 1:** Characteristics of the study group: study population of 73 eyes of 38 patients, divided into patients and eyes, diagnosed with childhood-onset, adamantinomatous craniopharyngioma (CP) before surgery and recruited in the study. Categorical variables are presented as n (%).

Craniopharyngioma (CP) Patient Characteristics	Study Cohort (Patients)	Study Cohort (Eyes)
	n (%) 38 (100%)	n (%) 73 (100%)
Sex, female/male	16 (42%)/22 (58%)	31 (42%)/42 (58%)
Place of birth, city/country	28 (74%)/10 (26%)	53 (73%)/20 (27%)
Mean age at CP diagnosis, years (range)	8.5 (1.9–16)	
Mean age at OCT examination, years (range)	10.3 (4–17)	
The reason for requesting diagnostics		
Headache	17 (45%)	32 (44%)
Visual impairment	10 (26%)	19 (26%)
Short stature	3 (8%)	6 (8%)
Symptoms at CP diagnosis		
Headache	26 (68%)	51 (70%)
Vomiting	17 (45%)	32 (44%)
Impaired visual acuity	22 (58%)	41 (56%)
Field of vision restriction	7 of 23 (30%)	14 of 43 (33%)
Optic disc oedema	12 (32%)	22 (30%)
Atrophy of the optic nerve disc	4 (11%)	7 (10%)
Strabismus	9 (24%)	17 (23%)
Double vision	2 (5%)	4 (5%)
The blindness of one eye	2 (5%)	2 (3%)
Drowsiness/disturbance of consciousness	16 (42%)	31 (42%)
Apathy	14 (37%)	27 (37%)
Epileptic seizure	5 (13%)	10 (14%)
Loss of consciousness	0	0
Memory disorders	4 (11%)	8 (11%)
Neurological symptoms	10 (26%)	19 (26%)
Growth retardation	16 (42%)	31 (40%)
Delayed puberty	3 of 9 at pubertal age (33%)	6 of 18 at pubertal age (33%)
GH deficiency	5 of 6 (83%)	10 of 12 (83%)
Hypothyroidism	13 (34%)	25 (34%)
Adrenal insufficiency	6 of 24 (25%)	12 of 49 (24%)
Arginine vasopressin deficiency	3 (8%)	6 (8%)
Hyperprolactinemia	6 of 26 (23%)	12 of 50 (24%)

**Table 2:** Characteristics of the study group study population of 73 eyes of 38 patients, divided into patients and eyes, with childhood-onset, adamantinomatous craniopharyngioma (CP) at diagnosis and after surgery, and recruited in the study. Categorical variables are presented as n (%).

Craniopharyngioma (CP) Patient Characteristics	Study Cohort (Patients)	Study Cohort (Eyes)
	n (%)	n (%)
Tumor location		
Intrasellar and suprasellar	24 (63%)	46 (63%)
Suprasellar	14 (37%)	27 (37%)
Intrasellar	0 (0)	0 (0)
Median tumor volume, cm^3^ (range)	35.2 (1.8–213.7)	
Median maximum tumor diameter, mm (range)	44.2 (19–98)	
Tumor morphology solid/cystic	4 (11%)/34 (89%)	7 (10%)/66 (90%)
Calcifications	37 (97%)	71 (97%)
Invading the third ventricle	29 (76%)	55 (78%)
Cavernous sinus infiltration	2 (5%)	4 (5%)
Hydrocephalus	16 (42%)	29 (40%)
Ventriculoperitoneal shunt	6 (16%)	12 (16%)
Bifrontal craniotomy/transcortical-transforaminal craniotomy	31 (87%)/5 (13%)	63 (86%)/10 (14%)
Degree of surgical resection (gross total resection/subtotal resection)	23 (61%)/15 (39%)	46 (63%)/27 (37%)
Rosenthal fibers in histopathology examination	5 (13%)	9 (12%)
SIADH after surgery	20 (53%)	39 (53%)
The end of AVD after surgery	4 (11%)	8 (11%)
Progression	5 (13)	9 (12%)
Recurrence	4 (11%)	7 (10%)
Reoperation	7 (8%)	13 (18%)
Radiotherapy	10 (24%)	19 (26%)
Memory disorder after surgery	14 (37%)	27 (37%)
Hyperfagia after surgery	19 (50%)	37 (51%)

SIADH = syndrome of inappropriate secretion of antidiuretic hormone.

**Table 3:** Impact of clinical symptoms before and after surgery on RNFL thickness in pediatric patients with craniopharyngioma. Statistically significant differences are presented for each RNFL sector (avgRNFL—average retinal nerve fiber layer; supRNFL—superior RNFL; infRNFL—inferior RNFL; TU—temporal upper sector; TL—temporal lower sector; IN—inferior nasal sector; IT—inferior temporal sector; SN—superior nasal sector; ST—superior temporal sector; NL—nasal lower sector; NU—nasal upper sector) depending on the presence of clinical factors before and after surgery. Values represent sectors with significant RNFL thinning (*p* < 0.05).

Clinical Factor	RNFL Parameter	Effect	Comparison of Medians	*p*-Value
Age below 5 years at the time of diagnosis	IN	Thinner when the diagnosis below 5 years of age	79 μm vs. 121	0.02
	NU	Thinner when the diagnosis below 5 years of age	56 μm vs. 96 μm	0.02
Place of birth, city/country	infRNFL	Thinner when born in the country	74 μm vs. 85 μm	0.03
	IT	Thinner when born in the country	112 μm vs. 130 μm	0.03
	IN	Thinner when born in the country	78 μm vs. 130 μm	0.04
	NU	Thinner when born in the country	29 μm vs. 37 μm	0.04
	SN	Thinner when born in the country	79 μm vs. 90 μm	0.004
Optic disc oedema (yes/no)	avgRNFL	Thinner with optic disc oedema	75 μm vs. 85 μm	0.02
	supRNFL	Thinner with optic disc oedema	78 μm vs. 87 μm	0.03
	infRNFL	Thinner with optic disc oedema	74 μm vs. 83 μm	0.03
	IN	Thinner with optic disc oedema	91 μm vs. 112 μm	0.04
	NL	Thinner with optic disc oedema	52 μm vs. 67 μm	0.04
Delayed puberty before surgery	avgRNFL	Thinner when delayed puberty before surgery	75 μm vs. 91 μm	0.005
	supRNFL	Thinner when delayed puberty before surgery	77 μm vs. 92 μm,	0.007
	infRNFL	Thinner when delayed puberty before surgery	74 μm vs. 90 μm	0.007
	ST	Thinner when delayed puberty before surgery	109 μm vs. 129 μm	0.03
	TU	Thinner when delayed puberty before surgery	62 μm vs. 78 μm	0.007
	IT	Thinner when delayed puberty before surgery	101 μm vs. 135 μm	0.007
	NL	Thinner when delayed puberty before surgery	52 μm vs. 67 μm	0.02
	SN	Thinner when delayed puberty before surgery	81 μm vs. 91 μm	0.02
Arginine vasopressin deficiency (AVD) before surgery	avgRNFL	Thinner when AVD before surgery	77 μm vs. 98 μm	0.02
	supRNFL	Thinner when AVD before surgery	78 μm vs. 102 μm	0.04
	infRNFL	Thinner when AVD before surgery	76 μm vs. 95 μm	0.006
	IN	Thinner when AVD before surgery	78 μm vs. 119 μm	0.04
	NU	Thinner when AVD before surgery	57 μm vs. 78 μm	0.04
	SN	Thinner when AVD before surgery	81 μm vs. 123 μm	0.008
	ST, TU, TL, IT, NL	Thinner when AVD before surgery	n.s.	n.s.
Growth hormone deficiency (GHD) before surgery	avgRNFL	Thinner when GHD before surgery	73 μm vs. 96	0.03
	infRNFL	Thinner when GHD before surgery	66 μm vs. 91 μm	0.03
	IN	Thinner when GHD before surgery	63 μm vs. 112 μm	0.03
	NL	Thinner when GHD before surgery	49 μm vs. 72 μm	0.03
	NU	Thinner when GHD before surgery	58 μm vs. 83 μm	0.03
	SN	Thinner when GHD before surgery	83 μm vs. 127 μm	0.03
Hyperprolactinemia before surgery	infRNFL	Thinner when hyperprolactinemia is present before surgery	66 μm vs. 78 μm	0.03
	IN	Thinner when hyperprolactinemia is present before surgery	64 μm vs. 80 μm	0.03
	NL	Thinner when hyperprolactinemia is present before surgery	43 μm vs. 53 μm	0.003
Hypothalamic involvement [32]	TL	Thinner when no hypothalamic involvement	34 μm vs. 50 μm	0.001
	NL	Thinner when no hypothalamic involvement	42 μm vs. 56 μm	0.04
SIADH after surgery	NL	Thinner when SIADH occurs after surgery	49 μm vs. 61 μm	0.01
The end of AVD after surgery	IT	Thinner when continuous AVD after surgery	98 μm vs. 115 μm	0.04
Memory disorder after surgery	TL	Thinner when there is a memory disorder after surgery	55 μm vs. 48 μm	0.03
Hyperfagia after surgery	IN	Thinner when hyperphagia occurs after surgery	73 μm vs. 90 μm	0.002

SIADH = syndrome of inappropriate secretion of antidiuretic hormone; n.s. = not significant.

**Table 4:** Proposed standard of ophthalmological care for patients with craniopharyngioma used at Children’s Memorial Health Institute in Warsaw, Poland.

At the Time of Diagnosis:
1. Assessment of distance and near visual acuity with the best corrected refractive error (best corrected visual acuity). In patients with visual acuity less than 0.1–VEP (visual evoked potentials), in the rest of patients as far as possible and as indicated. In preverbal children, VEP assessment
2. Examination of the anterior segment and fundus
3. Visual field examination in children who cooperate well—in all children over 8 years of age, in younger children, an attempt should be made to perform the examination
4. OCT examination with assessment of RNFL, GCC and GCL
Frequency of examinations:
At the time of diagnosis
2. Before surgery—only if more than 7 days have passed between diagnosis and surgical treatment
3. 7–10 days after surgery—earlier if there are indications or worrying symptoms
4. 3 months after surgery
5. Then every 6 months

## Data Availability

The data presented in this study are available on request from the corresponding author. The data are not publicly available due to ethical restrictions and the need to protect patient confidentiality.
